# A modified candy-plug technique to occlude false lumen in aortic dissection

**DOI:** 10.1186/s42155-022-00331-0

**Published:** 2022-10-29

**Authors:** Erik Palm, Antti Valtola, Hannu Manninen, Petri Saari

**Affiliations:** 1 Department of Radiology, Kuopio University Hospital, Puijonlaaksontie 2, 70210 Kuopio, Finland; 2 Department of Cardiothoracic Surgery, Kuopio University Hospital, Heart Center, Puijonlaaksontie 2, 70210 Kuopio, Finland; 3 University of Eastern Finland, Yliopistonranta 1, 70210 Kuopio, Finland

**Keywords:** Candy-plug, Aortic dissection, False lumen enhancement, False lumen occlusion

## Abstract

**Purpose:**

Aim of this technical note article is to introduce a modified, novel way to custom create a candy-plug (CP) device to endovascularly occlude false lumen. The technique is illustrated by a patient case with significant backflow to false lumen (FL). The patient had already undergone surgical repair of the ascending aorta, aortic arch and subsequent TEVAR procedure down to the ostium of the celiac trunk because of type A aortic dissection, but the descending thoracic aorta continued to dilate due to backflow to the FL from an uncovered tear at the level of the renal arteries.

**Materials and methods:**

We modified a Gore Excluder 36–45 mm aortic extender (W. L. Gore & Associates, Inc. Flagstaff, USA) endoprosthesis into a CP device that was subsequently positioned under local anesthesia into the FL of the distal descending thoracic aorta.

**Results:**

In 1 month control the backflow to false lumen had ceased and the aorta had decreased in diameter from 69 to 66 mm, FL from 37 to 34 mm, true lumen (TL) remained the same 32 mm.

**Conclusion:**

We describe a modified, effective candy-plug technique to occlude retrograde false lumen filling in aortic dissection.

## Introduction

According to recent guidelines, TEVAR (Thoracic Endovascular Aortic Repair) is recommended as a first-line treatment for complicated chronic type B aortic dissection (Riambau et al., 2017). However, total thrombosis of the FL occurs only in 40% of cases after closing the primary entry tear with TEVAR (Kusagawa et al., 2005). Persistent flow is associated with FL growth, threat of rupture and failure of TEVAR. During the last few years embolization of the distal FL with CP technique has shown promising results in facilitating favorable remodeling of FL. Home-made and commercially available CP devices have been introduced after the original introduction of this technique by Kölbel et al. in 2013 (Kölbel et al., 2013; Marone et al., 2017; Wu et al., 2018). Ogawa et al. described home-made CP using partly ligated GORE Excluder aortic extender (Ogawa et al., 2016). This procedure must be completed by closing the central channel with an Amplatzer plug. Commercially available 2nd generation CP are available as well as an option. We describe an alternative technique to prepare and deliver a CP made of Gore Excluder aortic extender (Gore Excluder, W. L. Gore & Associates, Inc. Flagstaff, USA) without the need for embolization of the central lumen.

## Case report

Patient is a 36-year-old man, who suffered a type A aortic dissection in September 2013 at the age of 28 years. The patient complained of right sided crushing jaw pain, chest pain, numbness and loss of muscle strength in the left leg. Acute type A aortic dissection with dilated ascending aorta was diagnosed on CT-angiography (CTA). Emergency repair of the ascending aorta with Bentall technique, hemiarch and proximal part of brachiocephalic trunk with tubular prostheses was performed. This support is also supported by the latest literature (Malaisrie et al., 2021). Genetic testing for an HTAD (hereditary thoracic aortic disorder) was scheduled right after primary operation but did not take place until 2020. The testing revealed no other hereditary disorders than MYH11 gene mutation.

The patient recovered well from the primary surgery. CTA 6 days after surgery revealed dissection distal to the left subclavian artery (LSA) with narrow TL which on day 8 was almost completely collapsed (TL 2 mm, FL 28 mm). However, this situation resolved without interventions.

The patient was lost to follow-up between 2015 and 2020 due to unknown reasons even though CTA examinations were scheduled. Finally, in December 2020 a routine follow-up aortic MRA demonstrated an increase of the proximal descending aorta from year 2015 up to 58 mm, calculated rate about 2.5 mm/year. TL diameter was 9 mm, FL 49 mm. The patient had developed a grade III/IV mitral valve regurgitation and the left ventricle had begun to dilate.

In March 2021, the aortic arch was reconstructed with frozen elephant trunk technique (Thoraflex® 32-30x100mm) with concomitant mitral and tricuspid valve repairs. The LSA was not reconstructed due to technical difficulties. CTA on postoperative day 5 showed significant backflow to FL. That is why carotid-subclavian bypass operation and subsequent TEVAR (proximal TAG 40–40-200 mm, distal TAG 37–37-100 mm, W. L. Gore & Associates, Inc. Flagstaff, USA) of the descending thoracic aorta was performed on day 6. On preoperative CTA it was noted that there are 2 pairs of prominent, 2 mm in diameter, intercostal arteries at the level and about 15 mm proximal to the ostium of the truncus celiacus and the decision was made to leave those intercostal arteries uncovered to minimize the risk of spinal ischemia. The patient was discharged from ICU on day 7 and to regional hospital on day 12.

In April 2021, continued filling of the FL most likely from the tear at the level of the renal arteries was noted on CTA, proximal descending aorta had expanded to 60 mm from preoperative 58 mm. By November 2021 it had grown to 69 mm (FL 37 mm, TL 32 mm). At this point the decision to treat FL backflow with custom made CP device was made. Thoracolaparotomy was the other option considered together, however the significantly less invasive endovascular approach with the CP device was chosen. The option to perform thoracolaparotomy would still remain should the CP fail for any reason. The procedure the treatment options were thoroughly explained to the patient and an informed consent was obtained.

## Materials & methods

### Candy-plug procedure

**Fig. 1:**
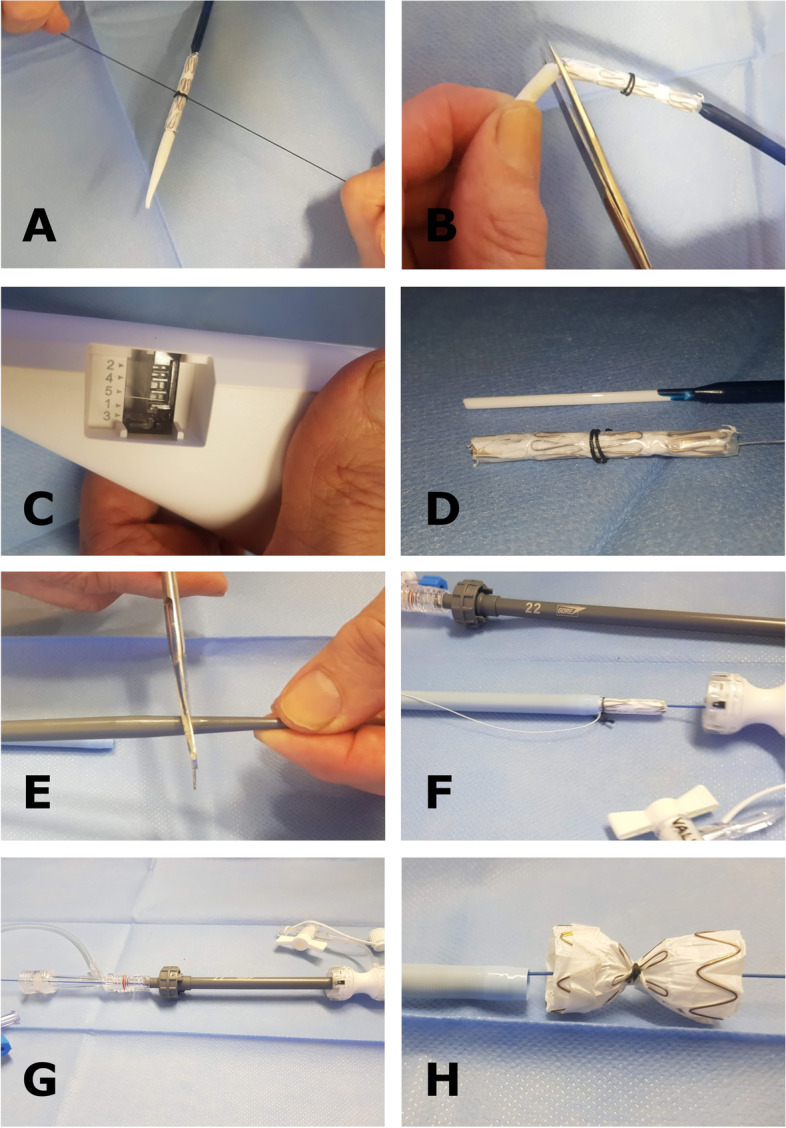
**A** A tight surgical knot was made between the centermost stent struts of the 36/45 mm aortic extender endoprosthesis (Gore Excluder, W. L. Gore & Associates, Inc. Arizona, USA). **B** The tip of the delivery catheter was bent so that the wiring was visible and cut. **C** The deployment wire was cut from the proximal end after opening the stent graft handle. **D** The graft with the wire was removed from the device. **E** Both the 18F and 22F dilator tips were cut. **F** The 18F dilator was placed back into the 18F sheath, in contact with the stent graft leaving the deployment wire outside the sheath. **G** The 18F sheath, dilator and stent graft were inserted as a package into the 22F sheath. **H** Demonstration of the opened CP after the 22F introducer sheath was retracted

**Fig. 2:**
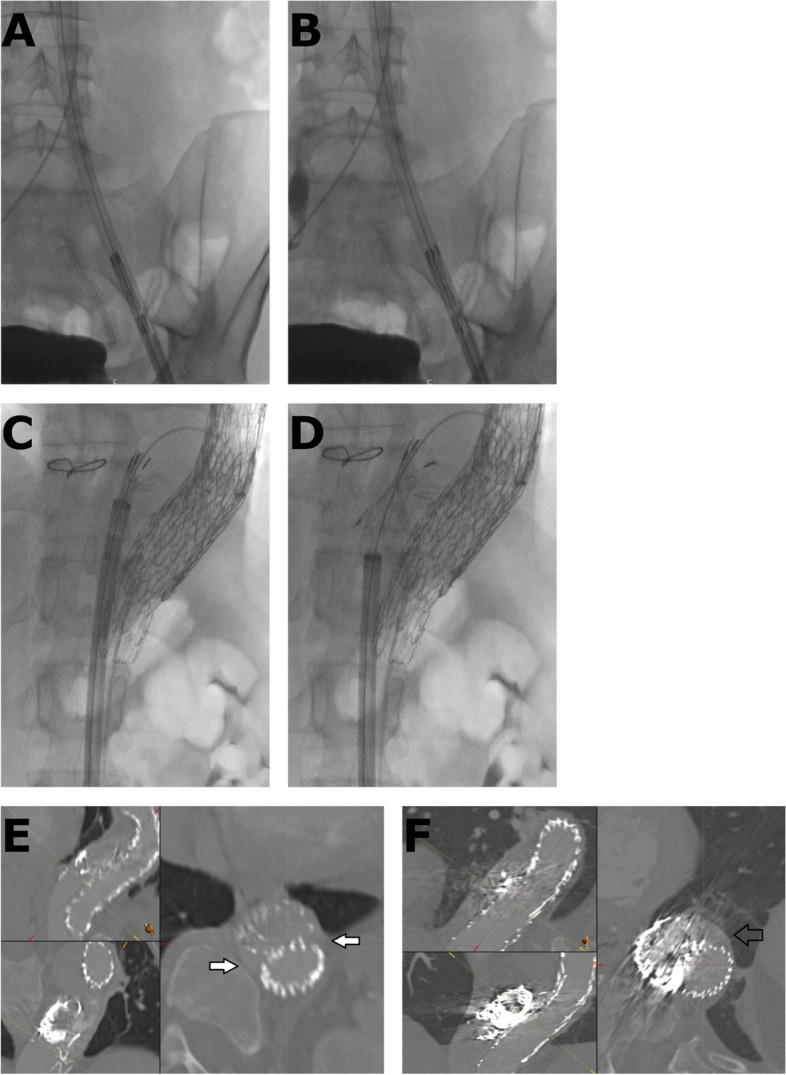
**A** and **B** The CP was advanced under fluoroscopy control close to the tip of the 22F sheath and opened by pulling from the deployment wire. Notice the CP closed in image A and expanded in image B inside the introducer sheath. **C** and **D** The CP was advanced within the sheath into the proper place and opened by retracting the 22F sheath accordingly. **E** and **F** Gutters at the distal end (**E**) and waist (**F**) of the CP after additional coiling

**Fig. 3:**
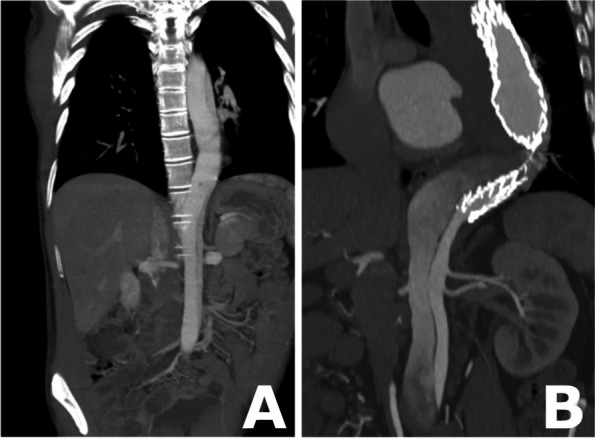
**A** Coronal CT image of the descending aorta of the patient after the surgery in 2013. **B** Coronal CT image of the descending aorta of the patient when the decision to apply the CP was made in 2021

**Fig. 4:**
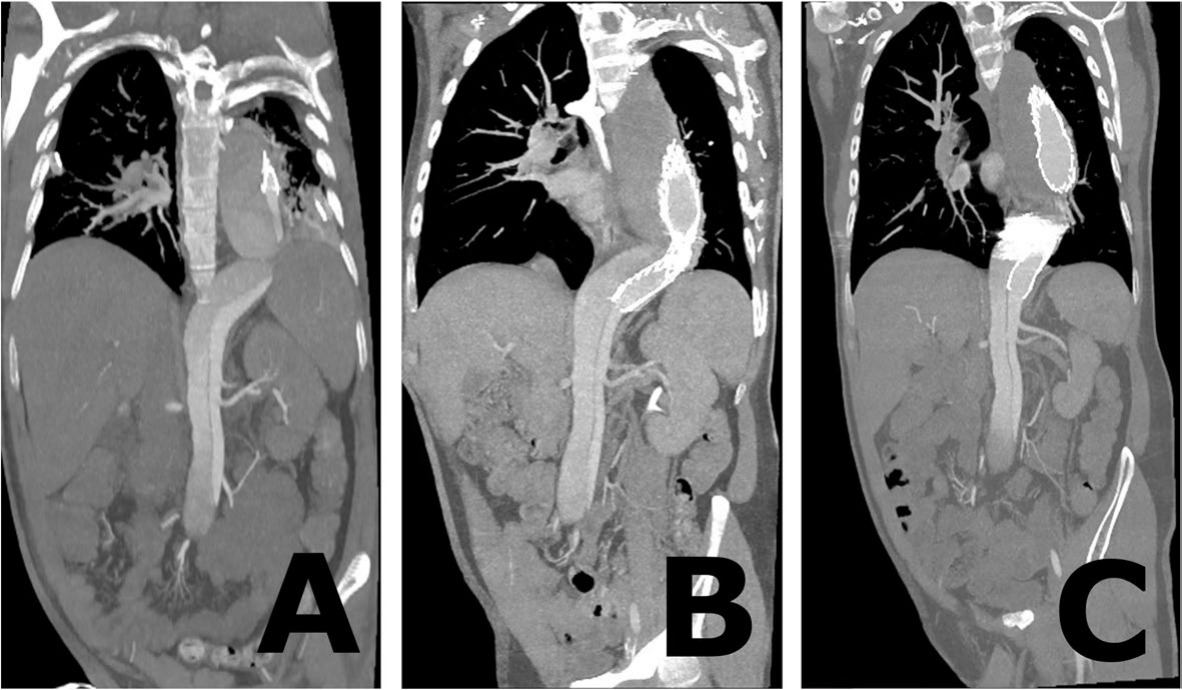
**A** Coronal CT image before extension TEVAR showing filling of the FL. **B** Coronal CT image after extension TEVAR showing continuous filling of the FL. **C** Coronal CT image after extension TEVAR and CP procedure showing significantly reduced filling of the FL

The procedure was performed under local anesthesia with continuous sedation. Access was gained via percutaneous right and left common femoral artery (CFA) under ultra-sound and fluoroscopy guidance. A 5F 11 cm sheath (Cordis Corporation, Miami Lakes, USA) and 5F STR imaging catheter (Cordis Corporation, Miami Lakes, USA) were advanced from the right CFA into the previous TEVAR. A 65 cm 22F introducer sheath (DrySeal, W. L. Gore & Associates, Inc. Flagstaff, USA) was placed from the left CFA into the abdominal aorta over a stiff guide wire (Back-up Meier, Boston Scientific Corporation, Marlborough, USA). At first stage a 37–37-100 mm thoracic endograft (Gore TAG, W. L. Gore & Associates, Inc. Flagstaff, USA) was placed as a continuation from the previous aortic stent graft and the distal end landed close to coeliac trunk. After that, the 22F sheath was navigated into the FL through the entry at the level of the renal arteries over Amplatz Super Stiff 0.035-in. guide wire. We prepared the CP device from tubular 36–45 mm aortic extender endoprosthesis (Gore Excluder, W. L. Gore & Associates, Inc. Flagstaff, USA). A tight surgical knot with standard surgical knot technique using thick braided nylon suture to minimize the risk of knot tear (Surgilon®, braided nylon) was made in-between the centermost stent struts (Fig. 1A). After that the tip of the delivery catheter was bent so that the wiring was visible and cut (Fig. 1B). Then the deployment wire was cut from the proximal end after opening the stent graft handle (Fig. 1C). The graft with the wire was removed from the device (Fig. 1D). The stent graft was then placed within the 18F sheath (DrySeal, W. L. Gore & Associates, Inc. Flagstaff, USA) at the distal end. Both the 18F and 22F dilator tips were cut (Fig. 1E). The 18F dilator was placed back into the 18F sheath, in contact with the stent graft leaving the deployment wire outside the sheath (Fig. 1F). The 18F sheath, dilator and stent graft were then inserted as a package into the 22F sheath which is already positioned into the FL (Fig. 1G). The stent graft was pushed into the 22F sheath with the 18F dilator and sheath which were then removed and replaced by the 22F cut dilator. The CP was advanced under fluoroscopy control close to the tip of the 22F sheath and opened by pulling from the deployment wire (Fig. 2A and B). The CP was then advanced within the sheath into the proper place and opened by retracting the 22F sheath accordingly (Figs. 1H, 2C and D). Along the waist of the CP some coils (Ruby Coil, Penumbra, Inc. Alameda, USA; Target XL® 360 Standard, Stryker Neurovascular, Fremont, USA) were delivered from a microcatheter to enhance thrombosis of the FL and especially the gutter around the CP (Fig. 2E, white arrows, F, empty arrow). Finally, balloon dilation with aortic balloon (Tri-Lobe 26–45 mm, W. L. Gore & Associates, Inc. Flagstaff, USA) was performed inside TEVAR prosthesis at the site of the CP. 3 days post-operatively, a CTA was performed and although the plug had expanded perfectly, significant backflow into the FL was still noted so an additional coiling procedure was performed. At 1 month CTA the backflow to FL had disappeared and aortic diameter had decreased in diameter from 69 to 66 mm, FL from 37 to 34 mm, true lumen (TL) remained the same 32 mm Figs 3 and 4.

## Discussion

Since Ogawa described the candy-plug technique using modified Gore Excluder extender, other investigators have applied the method successfully in elective interventions (Wu et al., 2018; Kotani et al., 2017) and also in acute aortic ruptures (Lin et al., 2019; Morisaki et al., 2019). The prepared CP is advanced to the desired position with the delivery catheter of the endoprosthesis. The removal of the delivery catheter is not always uneventful because the extender might get caught on the nosecone during retraction (Ogawa et al., 2016). Moreover, the central channel must be closed with an Amplatzer plug. This possesses the risk of CP moving inadvertently. The investigators also report that embolization of the central channel with Amplatzer plug was incomplete in two out of three patients. Even when not complicated this additional phase prolongs the procedure. Commercially available 2nd generation CP devices do not need occlusion of the central canal, however their ordering time is considerable, too long in some cases and thus we opted for our own custom made technique that offers the same benefits and is free from the before mentioned caveats.

Van der Steenhoven et al. (van der Steenhoven et al., 2011) first described the upside-down delivery of Gore Excluder iliac leg to expand utility of tapered leg prostheses to certain anatomies. Our modification of preparation of the CP is similar except that we ligated the waist of the endograft in the middle. The preparation and delivery of the CP to a desired position was quick and easy.

We completed the candy-plug placement with coil embolization of the FL gutters because partial contrast agent filling of the FL was detected on CT done 3 days after the original intervention. It is unlikely in our case that total thrombosis of FL might have occurred later, as reported by Ogawa (Ogawa et al., 2016), because of the rapid growth of the aneurysm. Also, other investigators have reported coiling in case of incomplete thrombosis of FL (Eleshra et al., 2019; Rohlffs et al., 2017). The other option would have been to use multiple CP devices, but we opted toward coiling, because the CP had expanded well and thus created a rather confined space around the waist of the CP to which we expected to be able to deliver significant amount of coils so they would facilitate thrombosis and stop the retrograde filling of the FL. Commercially available CP devices are an option, however the ordering time is a risk which we were reluctant to take and thus opted for a custom made device that is easy to make and has the same benefit of not having to occlude the central canal.

There are some drawbacks in our technique. After preparation of CP, it must be loaded into an introducer sheath that is 2 F larger than required by the plug if it is not dismounted from the delivery catheter of the aortic extender. Gore aortic extender is available up to a maximum diameter of 36 mm that might be too small in some cases.

## Conclusion

We conclude that our modification of candy-plug preparation and delivery is feasible and easy in order to achieve thoracic FL occlusion in chronic aortic dissection. However, one needs to take into account the possible need for additional embolization of the gutters to finalize the procedure.

## Data Availability

Not applicable.

## References

[CR1] Eleshra A, Kölbel T, Tsilimparis N (2019). Candy-plug generation II for false lumen occlusion in chronic aortic dissection: feasibility and early results. J Endovasc Ther.

[CR2] Kölbel T, Lohrenz C, Kieback A (2013). Distal false lumen occlusion in aortic dissection with a homemade extra-large vascular plug: the candy-plug technique. J Endovasc Ther.

[CR3] Kotani S, Inoue Y, Kasai M (2017). Modified 'candy-plug' technique for chronic type B aortic dissection with aneurysmal dilatation: a case report. J Cardiothorac Surg.

[CR4] Kusagawa H, Shimono T, Ishida M (2005). Changes in false lumen after transluminal stent-graft placement in aortic dissections: six years' experience. Circulation..

[CR5] Lin C-Y, Su I-H, Chu S-Y (2019). Modified candy-plug technique for rescue type B aortic dissection with false lumen rupture. Ann Vasc Surg.

[CR6] Malaisrie SC, Szeto WY, Halas M (2021). 2021 The American Association for Thoracic Surgery expert consensus document: Surgical treatment of acute type A aortic dissection. J Thorac Cardiovasc Surg.

[CR7] Marone EM, Leopardi M, Bertoglio L (2017). Original off-label endovascular solution to occlude false lumen rupture in chronic type B aortic dissection. Ann Vasc Surg.

[CR8] Morisaki A, Sohgawa E, Kishimoto N (2019). Candy-plug technique for ruptured chronic type B aortic dissection. Asian Cardiovasc Thorac Ann.

[CR9] Ogawa Y, Nishimaki H, Chiba K (2016). Candy-plug technique using an Excluder aortic extender for distal occlusion of a large false lumen aneurysm in chronic aortic dissection. J Endovasc Ther.

[CR10] Riambau V, Böckler D, Brunkwall J (2017). Management of descending thoracic aorta diseases: clinical practice guidelines of the European society for vascular surgery (ESVS). Eur J Vasc Endovasc Surg.

[CR11] Rohlffs F, Tsilimparis N, Fiorucci B (2017). The candy-plug technique: technical aspects and early results of a new endovascular method for false lumen occlusion in chronic aortic dissection. J Endovasc Ther.

[CR12] van der Steenhoven TJ, Heyligers JM, Tielliu IF (2011). The upside down Gore Excluder contralateral leg without extracorporeal predeployment for aortic or iliac aneurysm exclusion. J Vasc Surg.

[CR13] Wu IH, Chan CY, Luo CM, Wang SS (2018). Modified candy-plug device for aneurysmal false lumen occlusion in chronic type B aortic dissection. J Thorac Cardiovasc Surg.

